# Rabies Cases in the West of China Have Two Distinct Origins

**DOI:** 10.1371/journal.pntd.0004140

**Published:** 2015-10-20

**Authors:** Xiao-Yan Tao, Zhen-Yang Guo, Hao Li, Wen-Tao Jiao, Xin-Xin Shen, Wu-Yang Zhu, Simon Rayner, Qing Tang

**Affiliations:** 1 State Key Laboratory for Infectious Disease Prevention and Control, National Institute for Viral Disease Control and Prevention, Chinese Center for Disease Control and Prevention, Beijing, China; 2 Collaborative Innovation Center for Diagnosis and Treatment of Infectious Diseases, Hangzhou, Zhejiang, China; 3 Department of Medical Genetics, University of Oslo, Oslo, Norway; 4 Key Laboratory of Agricultural and Environmental Microbiology, Wuhan Institute of Virology, Chinese Academy of Sciences, Wuhan, Hubei, China; Atlanta Health Associates Inc., UNITED STATES

## Abstract

In China, rabies remains an ongoing threat to public health. Although control efforts have been effective in reducing the number of annual cases, the virus continues to spread into new areas. Tibet, Qinghai, Gansu and Ningxia in western China have, until recently, reported only a handful of events. However, since 2011, there have been increasing numbers of cases recorded in these areas. In this study, we report the collection and analysis of samples collected from these regions. We find that cases originate from two different sources. Strains collected from Gansu and Ningxia are closely related to the primary lineage associated with the current epizootic, whereas those from Tibet and Qinghai are related to the Arctic-like-2 lineage that is most commonly associated with wildlife cases in China. Thus, it appears that while the epizootic is beginning to encroach into Gansu and Ningxia, Tibet and Qinghai a significant number of rabies cases originate from wildlife.

## Author Summary

Overall, the number of annual cases of human rabies reported in China has been decreasing since 2007. However, some Western provinces, where few human cases have been reported in recent years, are beginning to see increasing incidence of rabies. Specifically, Ningxia, Qinghai and Gansu began to report human cases respectively from 2011, 2012 and 2013, while Tibet had its first laboratory confirmed dog rabies case in 2012. Consequently, as part of the national rabies surveillance program, we collected specimens from biting dogs or human saliva from suspected rabies cases in these areas, and after sequencing positive samples, performed a phylogenetic analysis based on the nucleoprotein gene complete sequences. Our results indicate that while Ningxia and Gansu rabies strains are very close to the lineage associated with most cases in mainland China, Tibet and Qinghai strains belong to the global Arctic-like-2 clade, which is typically associated with wild life in China and neighboring countries. Thus, it appears that, rabies reemergence in the west of China has two distinct origins, and Tibet and Qinghai rabies cases were isolated events rather than an indication of the ongoing epizootic in China.

## Introduction

Rabies continues to represent a serious threat in China. The ongoing rabies surveillance program in China has provided a comprehensive overview of the current outbreak, from the rapid increase in the annual number of cases at the beginning of the epizootic together with the gradual geographic spread of recorded events, to the subsequent drop in cases as control measures came into effect [1,2]. Data has also shown that, although the number of annual fatalities has fallen each year since 2007, new cases have been recorded in areas that were previously rabies free for many years [2,3].

**Fig 1:**
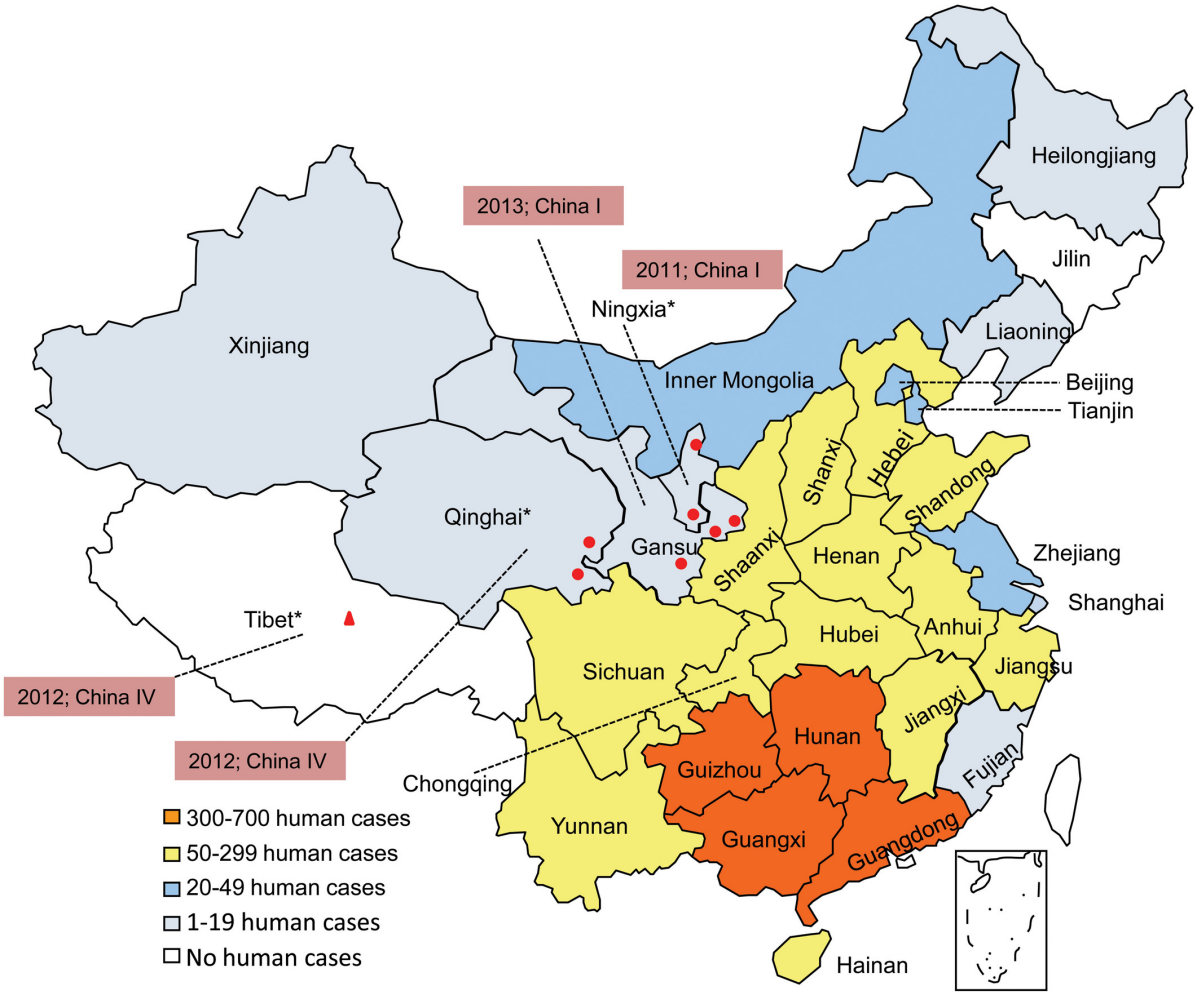
Geographical distribution of rabies cases in China, 2011–2013. Surveillance data indicates the highest numbers of cases are recorded in the south of the country with progressively fewer cases radiating away from this area. The provinces where the new samples were collected are marked with a“*”after the name, and the location and date of each case is shown together with the identified lineage. See main text for full details.

Tibet, Qinghai, Gansu and Ningxia, in Western China (Fig 1), are areas which have experienced minimal impact from rabies, with only a few cases reported from 1996 to 2010[1]. However, since 2011, Ningxia, Qinghai and Gansu all began to report human cases, and in Tibet, the first case of laboratory confirmed dog rabies was recorded in 2012. Here we report an analysis of the first case specimens collected in Tibet (dog), Qinghai, Gansu and Ningxia with the aim of investigating their origin.

## Materials and Methods

### Ethics statement

The program for collection of human saliva sample and dog brain specimens were approved by the Ethical Committee of the National Institute of Viral Disease Control and Prevention, China CDC, which is the national referral center for rabies diagnosis. Due to their medical condition, subjects were unable to provide consent once a rabies infection was suspected and so written informed consent was obtained in all cases from their relatives after death. Dog brain specimens were taken after suspected rabies death. No non-human primates were used in this study.

### Epidemiological data

Data on human rabies cases for each province between 2011 and 2014 from the whole of Mainland China were collected from the annual reports of the China CDC. The reporting methods and how cases were determined to be associated with rabies were the same as described previously [4,5].

### Specimen detection

The brain tissues of dogs were analyzed using the direct immunofluorescence assay (DFA) as described previously [6], and the liquid specimens such as human saliva were tested using the reverse-transcription polymerase chain reaction method (RT-PCR) [7]. All background data on human or animal specimens were provided by provincial or local CDCs.

### RT-PCR and sequencing

Total RNA extraction and cDNA synthesis procedures were the same as described previously [7]. The complete sequence of the N gene was amplified with two pairs of primers:N55F (5'ATGTAACACCTCTACAATGG 3', nt55~74) and N899R (5'GCCCTGGTTCGAACATTCT 3', nt881 ~ 899) primers were used for the first half segment with the locations of the primer sequences with respect to the full genome sequence of Pasteur virus(PV) strain (M13215);N644F and N1537R primers for the latter half were as described previously [6]. The N gene segments were amplified, the PCR products were purified and sequenced, sequencing results were assembled and gene coding regions for the nucleotide sequence were selected also as described previously [6,7]. The 4N gene sequences (1353bp) from Tibet, Qinghai, Gansu and Ningxia were submitted to GenBank, with accession numbers KC465379, KC465372, KM034905, KM034906.

### Phylogenetic analysis

**Fig 2:**
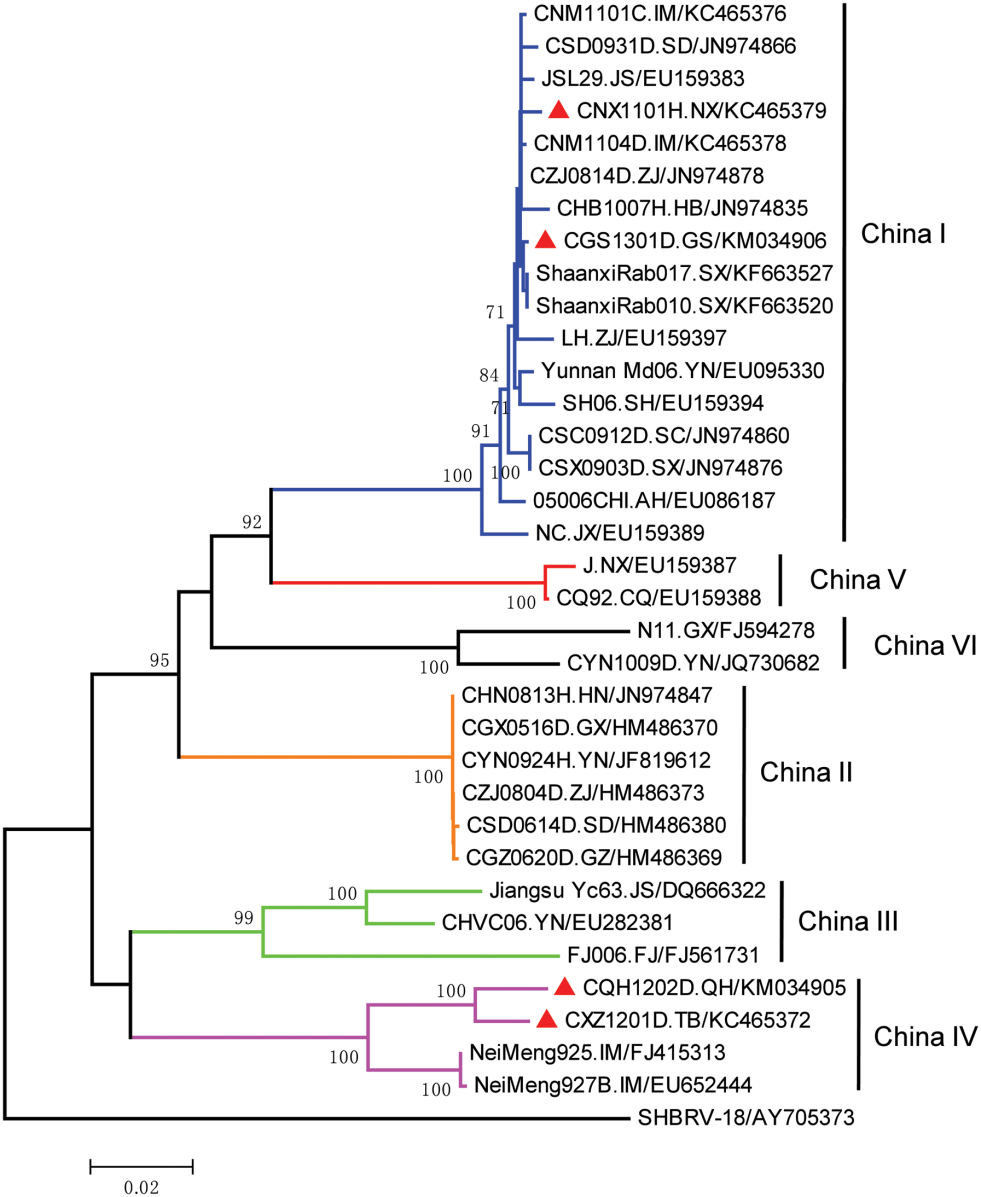
Neighbor-joining phylogenetic tree of Nucleoprotein gene sequence showing classification of specimens collected in western China during 2011–2013. Tree is based on the full region encoding the Nucleoprotein gene sequence (1353bp) and the American bat strain SHBRV-18is used as outgroup. Representative samples of each lineage were selected and samples reported here are marked with a red triangle. Branches are colored to indicate the six different lineages circulating in China. Specimen names are in the format (STRAIN.PROVINCE/GENBANK ACCESSION NO). Chinese provinces and regions are abbreviated with the following two letter codes:IM(Inner Mongolia), SD(Shandong), JS(Jiangsu), NX(Ningxia), ZJ(Zhejiang), HB(Hebei), GS(Gansu), SX(Shaanxi), YN(Yunnan), SH(Shanghai), SC(Sichuan), AH(Anhui), JX(Jiangxi), CQ(Chongqing), GX(Guangxi), HN(Hunan), GZ(Guizhou), FJ(Fujian), QH(Qinghai), TB(Tibet).

**Fig 3:**
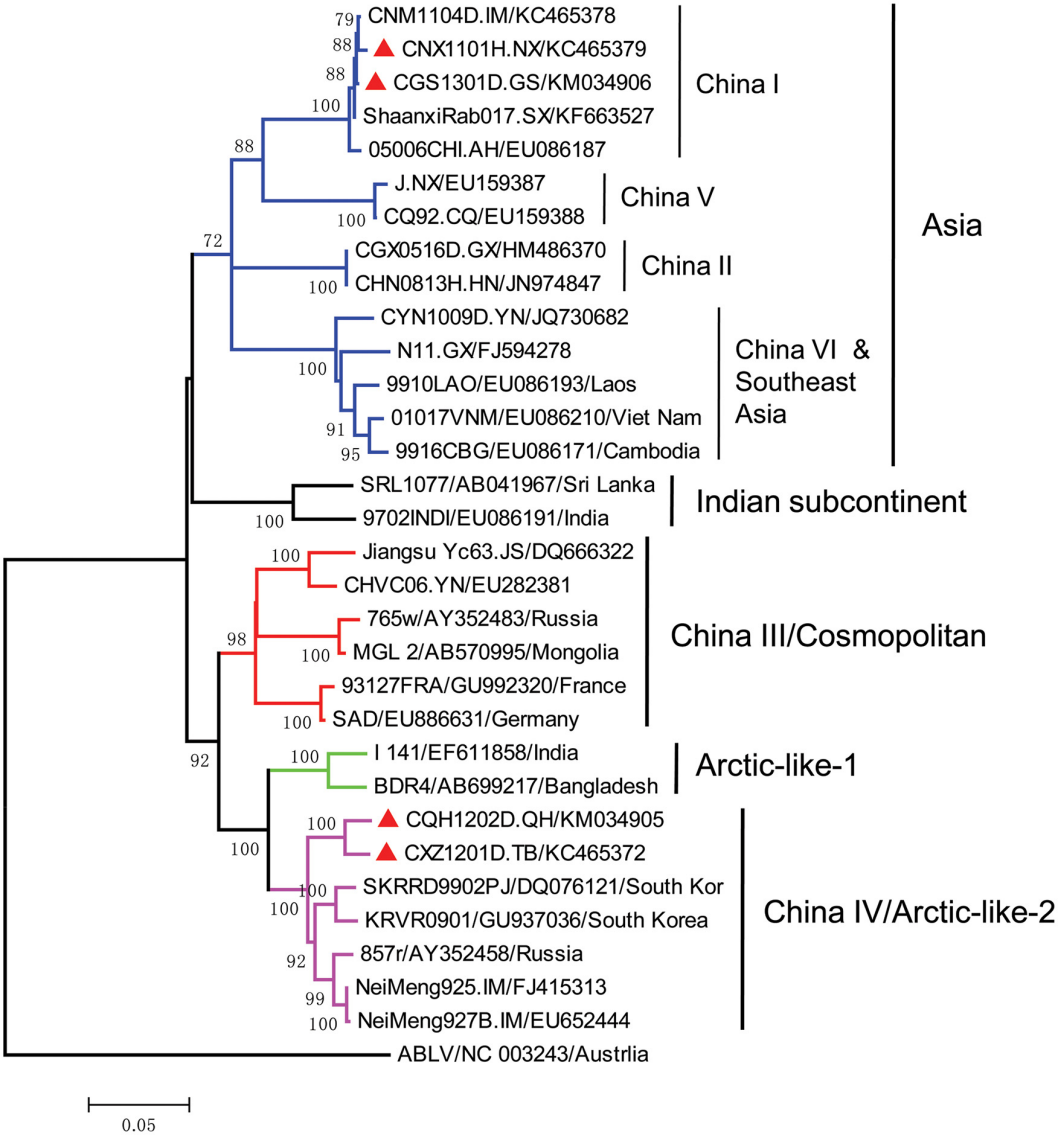
Neighbor-joining phylogenetic tree of the representative street strains from China and worldwide. Tree is based on the full region encoding the Nucleoprotein gene sequence and the Australia bat strain (ABLV) is used as an outgroup. Clades are colored to distinguish each of the recognized global lineages. The clades and specimen names from Fig 2 are shown, and the non-Chinese strains are in the FORMAT STRAIN/ GENBANK ACCESSION NO./COUNTRY. The strains sequenced in this study are marked with a red triangle.

The sequences were aligned using Clustal X v2.1 software [8]; Since the goal of the phylogenetic analysis was only to classify the strains in terms of the primary lineages circulating in China, phylogenetic reconstruction was performed using the MEGA5 software package with the Neighbour-Joining (NJ) method and 1000 bootstrap (BP) replicates [9]. Reference sequences that were representative of the major lineages both globally and within China were selected from GenBank based on earlier studies(Figs 2 and 3), with the exception of two Inner Mongolia strains CNM1101C(accession no. KC465376) and CNM1104D(accession no. KC465378), which are reported in this study.

### Accession numbers

The new sequenced N gene sequences in this study are with accession numbers KC465379, KC465372, KM034905, KM034906, KC465376 and KC465378.

## Results

### Background and phylogenetic analysis of rabies strains from Western China

Fig 1 shows the geographical location of rabies cases in Qinghai, Gansu and Ningxia and Tibet between 2011 and 2013: the recorded cases in Ningxia and Gansu are closer to Inner Mongolia and Shaanxi, where rabies has circled in recent years; the cases in Qinghai are in the eastern part of the province; and the dog specimen from Tibet was collected in the center of the region.

Phylogenetic analysis of the N gene sequence of the strains collected in Tibet, Qinghai, Ningxia and Gansu together with representative strains from each lineage is shown in Fig 3. The human specimen collected in Ningxia in 2011(CNX1101H.NX) was classified as China I and is most similar to the strain collected from Inner Mongolia the same year (CNM104D), but distinct from the specimen collected in Ningxia in 1985(J, China V). Given the observed dissemination of the China I lineage, it would suggest that the recent cases in the province are maybe a consequence of spillover from neighbouring provinces, rather than from a locally circulating strain that has been maintained in the region [1]. However, additional strains are needed to confirm this hypothesis. Similarly, strain CGS1301D.GS collected in Gansu in 2013 also clustered with the China I lineage, together with the strains collected in neighboring Shaanxi province in 2011 and 2012 (ShaanxiRab017, ShaanxiRab010), indicating a similar origin. However, the strains collected in Tibet and Qinghai are both classified as China IV/Arctic-like-2 and are grouped with the strains collected in Inner Mongolia, suggesting these cases are a consequence of spillover from wildlife. Thus, these recorded cases in these four regions appear to be disseminated from two distinct origins.

### Relationship of Chinese strains and world’s strains

Given the placement of the Tibet (CXZ1201D.TB) and Qinghai (CQH1202D.QH) specimens in the China IV/Arctic-like-2 lineage [10], we also generated a phylogenetic tree based on a set of global reference sequences (Fig 3). Both strains are placed in the Arctic-like-2 clade, together with canine or wildlife samples from South Korea, Russia and raccoon dog samples from Inner Mongolia.

## Discussion

Rabies is one of the notifiable infectious diseases in China and a comprehensive surveillance network exists for this disease. Analyses of surveillance data reveal a gradual dissemination of the virus across China, originating from the southern provinces and spreading out towards the northern and western provinces [11]. Results from earlier phylogenetic analyses based on the N and G gene sequences from almost all available Chinese street strains [1,12] indicate that the rabies virus circulating in China can be divided into 6 lineages (China I-VI). Analysis further indicates that the majority of recent cases are associated with China I and wildlife strains are generally classified as China IV (equivalent to Arctic-like-2 in global studies of rabies)[13,14].

In this study, we investigated four low incidence regions. Tibet and Qinghai are located in the Qinghai-Tibet Plateau, a sparsely populated and geographically remote region with climatic extremes. Between 1999 and early 2012 there were no rabies cases reported in Tibet. The first laboratory confirmed specimen (CXZ1201D.TB) was from collected from a stray dog that had bitten six villagers. In Qinghai, no cases had been recorded from 1987 to the end of 2012, when a human case was reported and confirmed by testing a brain specimen from a dog who had bitten this villager(CQH1202D.QH). A second human case was reported in 2013.

Ningxia reported two rabies cases in 2011, before this there were no recorded human cases for 8 years, and a saliva specimen of one patient (CNX1101H.NX) was confirmed positive by laboratory test. From 2011 to 2013, the number of human cases has increased each year and 8 human cases were reported in 2013. Similarly, Gansu had few rabies human cases reported for many years, with single cases reported in 1997, 2003 and 2009 and no cases reported from 2010 to 2012. However, 9 human cases were reported in 2013 and a brain specimen collected from a biting dog (CGS1301D.GS) was confirmed positive.

The surveillance system currently in place in these regions is an extension of the existing system that has been established in higher incident regions. This ensures a flow of information associated with clinical symptoms, case history and, where possible, laboratory tests from local health centers to regional CCDCs to the national infectious disease reporting system. In this way it has been possible to provide a comprehensive overview of the rabies situation in China in recent years [4,5]. Based on previous data, we expected to find the growing number of human cases in these regions associated with the new lineage, which would be an indication of the ongoing spread of the virus. However, the classification of the Tibet and Qinghai strains within the China IV / Arctic-like-2 lineage suggests that these cases are originating from wildlife. Also, while additional human cases have been recorded in Gansu (4) and Ningxia (14), to date, no further human cases have been observed in Tibet and Qinghai, suggesting the current epizootic has yet to become established these regions. However, this is based on a small number of strains and additional samples are needed to confirm or reject this hypothesis.

Conversely, classification of the sequenced strains from Gansu and Ningxia provinces as China I suggests that the two rabies human cases reported in Ningxia in 2011 (after an absence of 8 years) are a consequence of imported cases from neighboring provinces, this is also supported by the observation that all the reported human cases in Gansu were in districts that are close to Ningxia and Shaanxi (Fig 1).

In the absence of China I cases, it appears that Tibet and Gansu remain low incidence regions with respect to the current epizootic and the geographical remoteness of these regions as well as the low dog population density may be reasons why it is more difficult for the virus to become established. However, at the beginning of the 21st century, the CPC Central Committee implemented the “Strategic plan to promote coordinated regional development in Western China”. As a consequence, these regions have experienced rapid economic development and an improved infrastructure. The Qinghai-Tibet railway opened in 2006 and an improved highway system means the region is now better connected both internally and externally to neighboring provinces. Consequently, these provinces are experiencing an influx of migrants and more frequent movements, increasing the likelihood that rabies may be imported into the region. This underlines the urgency of implementing the control measures that have been put in place in previously high incidence provinces to prevent rabies becoming established in these low incidence regions. If this hypothesis is true then, with heightened awareness and effective vaccination programs it may still be possible to prevent the virus from becoming established in these regions.
